# Dysentery

**Published:** 1840-01

**Authors:** 


*Dysentery.*—From an interesting lecture by Dr. Gerhard at the Blockley Hospital, Philadelphia, we extract the following observations on the treatment of this disease.

"In the acute form of the disease, the treatment is sufficiently simple. The usual antiphlogistic means are required, with local applications to the inflamed mucous membrane, calculated to allay its irritability and remove its morbid secretions: these local remedies are narcotics and laxatives. In the practice of this hospital, especially during the present year, we rarely find it necessary to bleed. We give first a dose of castor oil, and then make use of the oily mixture. Calomel, either alone, or combined with opium or ipecacuanha, is by far the best remedy in severe cases; we sometimes also use ipecacuanha alone, or Dover's powder. In most cases mercurials are sufficient to effect a cure as soon as they produce ptyalism, when the symptoms of acute dysentery often cease at once. Half or a quarter of a grain of calomel, eve y two hours, will salivate in three or four days. It is usually combined with opium, to allay the griping, and prevent purging; or the pulv. ipecac. et opii may be employed in place of the opium, to effect the same objects. We have also frequently used ipecacuanha, either alone, or combined with opium or calomel. In the case of subacute dysentery before us, we have employed these articles, at times resorting to the acetate of lead, and various astringents, without much advantage: Dover's powder has produced the most benefit. In the acute case, we have administered half a grain of calomel, with three grains of Dover's powder, every two hours.

We rarely employ calomel as a *purgative* in this disease. We use it for a few days only, to produce its specific antiphlogistic effect—that is, until slight ptyalism is induced. If it is not then attended with good effects, it should be given up: a continuance of its use will do much injury, and tend to increase the ulceration of the bowels.

This is a peculiarity in the action of mercurials; in many acute imflammatory diseases, the advantages to be gained are when the point of salivation is reached, which is a test of the operation of the disease, and the system may then be regarded as saturated. I am quite convinced that if, from any peculiarity of the system, or from the disease assuming an unusual tendency to the spreading of the ulcerations, mercury should be administered after ptyalism has been produced without benefit, the patient is decidedly injured. The remedy is best adapted to the inflammatory forms of the disorder, and, as we shall presently see, is least fitted for the sloughing or malignant variety.

Of the particular remedies in dysentery, purgatives have been extensively employed. We have used many articles of this class; the best we find to be castor oil, which purges sufficiently to carry off the vitiated secretions, without producing much irritation. To prevent the oil from acting too harshly, and to lessen the irratability of the bowels, laudanum may be advantageously combined with it. The oleaginous mixture

11

is a good formula for their combination ; of this we may give half an ounce every two hours, till it begins to act on the bowels. Rhubarb will also answer well as a purgative, and when the active symptoms have declined, the spiced syrup answers better than any other remedy. Venesection is sometimes required in acute dysentery, when the pulse is strong and corded; but we have not found it necessary in any case which has occurred in this hospital during the present year. The epidemic character of the disease has not been of the violent inflammatory character, which is a cardinal point in the diseases of the mucous surfaces, and seems necessary to the perfect cessation of the disease. I would not have you to misunderstand me, the term, restoration of the secretions, has been much abused and used vaguely. It means simply, in this case, to bring about the natural secretions of mucus, &c., in place of the diseased ones of blood and lymph. A certain set of remedies tend directly to produce this effect, and by restoring the natural secretions, they not only prove that the disease is ceasing, but they contribute to its cessation by producing depletion in the most effectual way, that is, through the natural emunctories of the part. Cups and leeches to the abdomen, along the course of the colon, are also frequently advisable ; the latter may also be applied around the anus, for the purpose of drawing blood from the hæmorrhoidal vessels, and relieving the tenesmus. Warm fomentations are very often beneficially employed. But these measures, however important, cannot alone be relied on for the cure of the disase ; we must restore the secretions to their healthy condition. This is a principal, though not the only object for which we employ calomel, with opiates, &c. The action of opium in dysentery is peculiar: in the first place, it allays the local pain and general irritability; and secondly, it quiets the spasmodic movements of the intestine, and thereby facilitates the process of cicatrization. But it may likewise produce bad effects; it tends to lock up the bowels, and prevent the discharge of the morbid secretions. To obviate this disadvantage we seldom use it alone, but combine it with castor oil, calomel, or ipecac. It may sometimes, however, be employed singly, either at the commencement or towards the close of the disease ; but never during the height of the inflammation. Opium is also used by *injection*. In this city, opiate injections in dysentery have not been much employed till within the last few years ; and in the country their use is still very limited, but in this hospital we are in the habit of using them very largely. From twenty to forty drops of laudanum may be administered in this way, but not more, for dangerous consequences from time to time result from the frequent employment of large quantities of so powerful a narcotic, particularly when given by the rectum, in which mode of administration its action upon the brain is more irregular than when given in any other way. We usually inject twenty drops of laudanum mixed with a small portion of mucilage, every two, three, or four hours, according to the severity of the tenesmus, and the effects of the remedy; thus, if the stools cease, or if the mind becomes confused, dull, or the patient sleepy, its use should be suspended. There is still another way in which opium may be employed in dysentery; that is, by means of poultices sprinkled with laudanum, and applied to the abdomen.

Of the other remedies employed in dysentery, ipecacuanha, as we have already mentioned, is among the most useful. It is used either singly or combined with calomel or opium. A very effectual method of administering it, is in combination with extract of gentian and blue mass. This combination originated with Mr. Twining, and has been extensively and beneficially employed in India. It generally produces vomiting at first, but in a short time this effect ceases. We have tried it in one epidemic; its administration was followed by nausea and diaphoresis, and a considerable alleviation of the symptoms. It sometimes failed, but was generally successful. The proportions are, six grains of ipecacuanha, four of blue mass, and five of the extract of gentian.

Various other remedies have been employed in acute dysentery. They are principally depletions, such as saline purgatives, calomel in large doses, &c. These will doubtless answer in many of the ordinary cases of the disease.—*Ib.*

